# A Metabolomics approach for the diagnosis Of SecondAry InfeCtions in COVID-19 (MOSAIC): a study protocol

**DOI:** 10.1186/s12879-021-06832-y

**Published:** 2021-12-02

**Authors:** Gordan McCreath, Phillip D. Whitfield, Andrew J. Roe, Malcolm J. Watson, Malcolm A. B. Sim

**Affiliations:** 1 Institute of Infection, Immunity and Inflammation, Sir Graeme Davis Building, University Place, Glasgow, Scotland; 2 Glasgow Polyomics, University of Glasgow, Garscube Campus, Glasgow, Scotland; 3 School of Medicine, Dentistry and Nursing, College of Medical, Veterinary and Life Sciences, University of Glasgow, Glasglow, G12 8QQ Scotland

**Keywords:** Metabolomics, Secondary infection, COVID-19, Critical care

## Abstract

**Background:**

Critically ill patients with COVID-19 are at an increased risk of developing secondary bacterial infections. These are both difficult to diagnose and are associated with an increased mortality. Metabolomics may aid clinicians in diagnosing secondary bacterial infections in COVID-19 through identification and quantification of disease specific biomarkers, with the aim of identifying underlying causative microorganisms and directing antimicrobial therapy.

**Methods:**

This is a multi-centre prospective diagnostic observational study. Patients with COVID-19 will be recruited from critical care units in three Scottish hospitals. Three serial blood samples will be taken from patients, and an additional sample taken if a patient shows clinical or microbiological evidence of secondary infection. Samples will be analysed using LC–MS and subjected to bioinformatic processing and statistical analysis to explore the metabolite changes associated with bacterial infections in COVID-19 patients. Comparisons of the data sets will be made with standard microbiological and biochemical methods of diagnosing infection.

**Discussion:**

Metabolomics analyses may provide additional strategies for identifying secondary infections, which might permit faster initiation of specific tailored antimicrobial therapy to critically ill patients with COVID-19.

## Background

### COVID-19 and secondary infections

The novel coronavirus severe acute respiratory syndrome coronavirus 2 (SARS-CoV-2) continues to present a major healthcare burden globally, with over 170,000,000 confirmed cases and 3,500,000 deaths worldwide [1]. Coronavirus Disease 2019 (COVID-19) has a spectrum of severity, and while the vast majority of cases result in a minor self-limiting illness, approximately 5% of patients will become critically unwell with severe respiratory failure which can progress to sepsis and multiple organ failure [2, 3]. Despite recent advances in treatment strategies, the mortality rate for COVID-19 patients admitted to the intensive care unit (ICU) in Scotland remains high at around 35% [4, 5].

The incidence of secondary infections in hospitalised patients with COVID-19 appears relatively low with rates between 6 and 15% [6–8]. However the rates are significantly higher in critically ill patients and carry a mortality rate of around 50% [8–10]. The clinical features of COVID-19 such as pyrexia, cough and dyspnoea are non-specific and are also observed in bacterial pneumonia [11]. It can therefore be difficult to make a diagnosis of secondary infection by clinical means [12]. Biomarkers such as lactate, C-reactive protein (CRP) and procalcitonin have a well-established role in identifying septic patients who are at risk of further deterioration, but as of yet a specific biomarker to detect the presence of a secondary infection remains elusive [13–15]. As such, liberal use of broad spectrum antibiotics has been observed in critically ill COVID-19 patients [2]. The World Health Organisation (WHO) and the Surviving Sepsis Campaign both recommend initiating empirical antibiotics for all severe cases of COVID-19 [12, 16], whereas the National Institutes of Health (NIH) and the National Institute for Health and Care Excellence (NICE) suggest only starting antibiotics when there is a clear clinical suspicion of a secondary infection [17, 18]. Early initiation of antibiotic therapy has been shown to reduce mortality in bacterial sepsis [19], however unnecessary use of broad spectrum antibiotics increases the risk of side effects and promotes antimicrobial resistance [20]. Prompt diagnosis of a secondary infection and identification of the causative pathogen is therefore important in optimal management. Microbiological cultures can guide antimicrobial therapy, but these are time-consuming, and have a low sensitivity [21]. Alternative diagnostic strategies to improve sensitivity and provide rapid specificity would therefore be valuable.

All infections have the potential to cause major disruption to physiological processes, potentially leading to a degree of metabolic dysfunction [22, 23]. This can lead to alteration of the normal serum metabolome (all low molecular weight metabolites less than 1 kDa circulating in the bloodstream at a given point in time) [24]. The physiological response to an infection can result in depletion of certain important nutrients, while simultaneously causing accumulation of other toxic by-products [23]. As such, variation in the composition of the metabolome can be indicative of pathological processes occurring further upstream [24].

### Metabolomics

Metabolomics is a discipline which is gaining much traction as a potential diagnostic tool. Metabolomic analysis using techniques such as liquid chromatography–mass spectrometry (LC–MS) can be used to provide a metabolic profile of a patient. This acts as a ‘snapshot’ of the patient’s metabolome, providing a description of the metabolic state of a patient as a result of both genetic contributions and environmental factors [24].

Metabolomics has been investigated for aiding diagnosis of a wide variety of diseases, however in recent years particular focus has been given to its use in identifying biomarkers for infections and sepsis, with several candidate metabolites showing promise. Derangement of fatty acid metabolism has been observed during a septic response, with increased levels of acylcarnitines in septic patients compared with non-septic controls, as well as lower levels of lysophosphatidylcholines in septic shock patients who responded poorly to initial therapy [25–28]. Animal and human studies have shown that changes in levels of tricarboxylic acid cycle intermediates can occur in response to infection. Derangement of concentrations of citrate, malate and succinate have been observed, particularly in response to gram positive infections including *Staph. aureus* and *Strep. Pneumoniae* [29–31]. Increases in protein catabolism and amino acid degradation have also been reported during a septic inflammatory response. Multiple studies have shown depletion of the essential amino acid tryptophan leading to a detectable accumulation of the toxic metabolite kynurenine [25, 32, 33]. The biogenic amine trimethylamine-N-oxide (TMAO) has also shown promise as a biomarker of infection. Gut microflora metabolise quaternary ammonium compounds such as betaine and choline to trimethylamine (TMA), which is then converted to TMAO in the liver. TMAO levels are thus indirectly dependant on a functioning gut microbiome [34, 35].

The wide range of possibilities in terms of site of infection, organism type and physiological response means that the resulting metabolic changes will vary from patient to patient. As a result, a single biomarker is unlikely to be sufficient to diagnose a secondary infection. Consideration of groups, or panels of metabolites may be more sensitive for diagnosing secondary infections than individual biomarkers [36]. Moreover, different types of infections may produce a unique signature metabolic profile, thus potentially making identification of the specific organism possible [37].

### Study rationale

In critically ill patients with COVID-19, examination of metabolic profiles may permit characterisation of novel biomarkers. These could permit detection of specific underlying infective organisms more rapidly than traditional culture methods. This would allow for earlier initiation of targeted antibiotic therapy, potentially improving outcomes in critically ill patients with COVID-19.

### Aim

The primary aim of this study is to ascertain the diagnostic capability of metabolomics for identifying secondary infections in critically ill patients with COVID-19 compared with routinely collected markers of infection.

Secondary objectives will compare profiles between the following subgroups:

- Bacterial and fungal infections
- Gram positive and gram-negative infections
- Healthy controls and COVID-19
- Survivors and non-survivors
- Patients with and without septic shock

This study aims to determine the potential diagnostic capability of metabolomics in diagnosing secondary infections in critically ill patients with COVID-19. We hypothesise that metabolomic profiling of patients with COVID-19 may permit identification of those with secondary infections. As such it is hoped that metabolomic profiling may provide additional perspectives on the underlying pathogen, thus aiding targeted antimicrobial therapy.

## Methods

### Study design

A multi-centre prospective diagnostic observational study.

### Participating centres

- Queen Elizabeth University Hospital, UK
- Glasgow Royal Infirmary, UK
- Royal Alexandra Hospital, UK

### Participant selection

Inclusion criteria:

Patients over the age of 18 with a confirmed positive SARS-CoV-2 test in the previous 7 days who are admitted for at least level 2 care will be eligible for inclusion.

Exclusion criteria:

Patients will be excluded if they cannot provide informed consent directly or by their nearest relative if they are incapacitated. Pregnant patients will be excluded, as will those who are deemed to be end of life.

### Sample size rationale

Taking into consideration the number of intensive care admissions during the first wave of the COVID-19 pandemic, we aim to recruit 75–100 critically ill COVID-19 patients and 50 healthy volunteers within a period of 1 year.

The first patient was recruited in November 2020, and recruitment will continue until December 2021, or until a sufficient number of patients have been recruited to provide 50 healthy control samples and 300 critically ill COVID-19 samples.

Primary endpoint

- Comparison of metabolomic profiles of COVID-19 patients with and without secondary infection

Secondary endpoints

- Sensitivity and specificity of metabolomics in identifying secondary infections
- Comparison of profiles between bacterial and fungal infections
- Comparison of profiles between gram positive and gram-negative infections
- Identification of prognostic biomarkers through comparison of profiles between survivors and non-survivors, and those with and without septic shock as per the Sepsis-3 definition [22].

### Study procedures

Patients eligible for recruitment will be identified and approached by the local research team upon their admission to the critical care department. Eligibility will be assessed against the inclusion and exclusion criteria by a doctor with an up-to-date Good Clinical Practice certificate.

Informed consent will be obtained for all patients recruited to the study. Where possible, consent will be sought directly from the patient. Patients will be asked to sign two copies of the consent form: one which they will retain, and a second to be filed in their medical notes. The consent forms will be counter-signed by the researcher gaining consent. Where patients are incapacitated, their nearest relative will be approached for consent. This will be discussed either in person or by telephone. Patients who regain capacity after recruitment will be approached to discuss consent for ongoing participation in the study.

Recruited patients will have serial blood samples collected at three time points: Day 0, day 3 and day 10. In addition, a blood sample will be collected if participants show evidence of a secondary infection. This includes growth of organisms from microbial cultures, or clinical deterioration which results in a septic screen being sent by the clinical team.

Blood samples will be centrifuged within 1 h of sample collection. Serum will be aliquoted and frozen at – 80 °C for storage. Metabolites will be extracted in chloroform:methanol:water, 1:3:1, v/v/v solution. Untargeted metabolomic analysis will be undertaken using LC–MS in both positive and negative ion modes on a Thermo Orbitrap system interfaced to a Dionex UltiMate 3000 Rapid Separation LC platform with a zwitterionic polymeric hydrophilic interaction chromatography (ZIC-pHILIC) column and ammonium carbonate in water/acetonitrile gradient. The raw LC–MS data sets will be processed by in-house bioinformaticians through an established pipeline, which uses XCMS and MZMatch [38–40]. Core metabolite identifications will be validated by comparison of the chromatographic retention times and the mass to charge values (m/z) values against a panel of authentic standards.

### Clinical data

Relevant patient data will be extracted from clinical notes. Data will be collected by the local research team using standardised data collection forms. Completed forms will be transcribed to a password protected electronic spreadsheet kept on a secure server. Paper forms will then be stored securely in the site file within a locked office at the participating site. All data will be anonymised at site and a unique study number will be allocated to each patient.

The following clinical data will be collected:

- Age
- Sex
- Ethnicity
- Body mass index
- Pre-existing comorbidities
- Date of SARS-CoV-2 infection
- Hospital and critical care length of stay
- Survival outcome
- Organ support requirements
- Sequential Organ Failure Assessment (SOFA) score [22]
- Biochemical data including CRP, procalcitonin, white cell count and lactate
- Positive microbiological cultures—organism and site of infection
- Use of antibiotics, antivirals, corticosteroids and immune modulating drugs.

Common contaminants of blood cultures such as coagulase-negative *staphylococci*, diphtheroids, *Bacillus* species or *Propionibacterium* species will not be considered positive microbiological cultures unless they are cultured from serial samples and treatment is initiated.

### Planned analysis

Statistical analysis of the untargeted metabolomic data sets will be guided by in-house bioinformaticians and will consist of univariate- and multivariate analyses.

### Patient confidentiality

Patient confidentiality will be maintained throughout the study as per the Data Protection Act. Recruited patients will be assigned a unique study number. All data collection sheets, study reports and communication regarding the study will identify the patients by this study number and initials only.

### Adverse event reporting

This is an observational study, and so adverse events are unlikely to occur. Any adverse events or serious adverse events will be recorded on case report forms, and an annual summary will be given to the ethics committee and research and development office.

### Protocol deviation reporting

A variation of the approved protocol will be considered a protocol deviation. Any protocol deviations will be recorded on case report forms and reported to the sponsor.

### Publication policy

Authorisation for all publications will be sought from the study chief investigator and will be reviewed by the sponsor prior to publication.

## Discussion

The COVID-19 pandemic is a continually evolving situation, and the future remains uncertain. Despite successful vaccine rollout, ongoing community transmission and the emergence of variants with increased virulence make continued hospital and critical care admissions likely. As such, secondary infections are likely to continue to be an issue in COVID-19 patients. Moreover, the increasing use of immune modulating therapies as part of COVID-19 treatment could potentially increase rates of secondary infections further [41].

Metabolites identified in this study could be utilised as biomarkers of infection. These may be capable of detecting secondary infections earlier, helping to identify specific pathogenic strains and allowing for monitoring of response to treatment. This would allow for initiation of tailored antimicrobial therapy, reducing reliance on broad spectrum empirical antibiotics, thus helping to reduce rates of antimicrobial resistance.

### **Trial status**

The trial is currently open for patient recruitment.

## Data Availability

The anonymized datasets generated during the current study will be made available from the corresponding author on reasonable request.

## Abbreviations

COVID-19: Coronavirus disease 2019
CRP: C-reactive protein
ICU: Intensive care unit
LC–MS: Liquid chromatography–mass spectrometry
NICE: National Institute for Health and Care Excellence
NIH: National Institutes of Health
SARS-CoV-2: Severe Acute Respiratory Syndrome Coronavirus 2
SOFA: Sequential Organ Failure Assessment
TMA: Trimethylamine
TMAO: Trimethylamine-N-oxide
WHO: World Health Organisation
ZIC-pHILIC: Zwitterionic polymeric hydrophilic interaction chromatography

## References

[1] World Health Organisation. WHO Coronavirus Disease (COVID-19) Dashboard. 2020. Accessed 31 May 2021. https://covid19.who.int/.

[2] Zhou F, Yu T, Du R (2020). Clinical course and risk factors for mortality of adult inpatients with COVID-19 in Wuhan, China: a retrospective cohort study. Lancet.

[3] Guan W, Ni Z, Hu Y (2020). Clinical characteristics of coronavirus disease 2019 in China. N Engl J Med.

[4] Recovery Collaborative Group (2021). Tocilizumab in patients admitted to hospital with COVID-19 (RECOVERY): a randomised, controlled, open-label, platform trial. Lancet (London, England).

[5] Scottish Intensive Care Society. Scottish Intensive Care Society Audit Group report on COVID-19. Published 2021. Accessed April 29, 2021. https://beta.isdscotland.org/media/8302/2021-03-31_sicsag_report.pdf.

[6] Hughes S, Troise O, Donaldson H, Mughal N, Moore LSP (2020). Bacterial and fungal coinfection among hospitalized patients with COVID-19: a retrospective cohort study in a UK secondary-care setting. Clin Microbiol Infect.

[7] Goyal P, Choi JJ, Pinheiro LC (2020). Clinical characteristics of COVID-19 in New York City. N Engl J Med.

[8] Ripa M, Galli L, Poli A (2021). Secondary infections in patients hospitalized with COVID-19: incidence and predictive factors. Clin Microbiol Infect.

[9] Zhang H, Zhang Y, Wu J (2020). Risks and features of secondary infections in severe and critical ill COVID-19 patients. Emerg Microbes Infect.

[10] Bardi T, Pintado V, Gomez-Rojo M (2021). Nosocomial infections associated to COVID-19 in the intensive care unit: clinical characteristics and outcome. Eur J Clin Microbiol Infect Dis.

[11] Seaton RA (2021). Bacterial co-infection is unusual in noncritical COVID-19 cases, so we must reduce our antibiotic prescribing. Pharm J.

[12] Alhazzani W, Møller MH, Arabi YM (2020). Surviving Sepsis Campaign: guidelines on the management of critically ill adults with Coronavirus Disease 2019 (COVID-19). Intensive Care Med.

[13] Faix JD (2013). Biomarkers of sepsis. Crit Rev Clin Lab Sci.

[14] Danwang C, Endomba FT, Nkeck JR, Wouna DLA, Robert A, Noubiap JJ (2020). A meta-analysis of potential biomarkers associated with severity of coronavirus disease 2019 (COVID-19). Biomark Res.

[15] Vazzana N, Dipaola F, Ognibene S (2020). Procalcitonin and secondary bacterial infections in COVID-19: association with disease severity and outcomes. Acta Clin Belg.

[16] World Health Organisation. COVID-19 Clinical Management Living Guidance 25 January 2021. World Health Organization; 2021. WHO/2019-nCoV/clinical/2021.1

[17] National Institutes of Health. Coronavirus disease 2019 (COVID-19) treatment guidelines. NIH Bethesda, MD, USA. Published online 2020. 34003615

[18] National Institute for Health and Care Excellence. COVID-19 rapid guideline: managing COVID-19. 2021. Accessed June 16, 2021. https://www.nice.org.uk/guidance/ng191 34181371

[19] Seymour CW, Gesten F, Prescott HC (2017). Time to treatment and mortality during mandated emergency care for sepsis. N Engl J Med.

[20] Cox MJ, Loman N, Bogaert D, O’Grady J (2020). Co-infections: potentially lethal and unexplored in COVID-19. Lancet Microbe.

[21] Cheng MP, Stenstrom R, Paquette K (2019). Blood culture results before and after antimicrobial administration in patients with severe manifestations of sepsis: a diagnostic study. Ann Intern Med.

[22] Singer M, Deutschman CS, Seymour CW (2016). The third international consensus definitions for sepsis and septic shock (Sepsis-3). JAMA.

[23] Pravda J (2014). Metabolic theory of septic shock. World J Crit care Med.

[24] Holmes E, Wilson ID, Nicholson JK (2008). Metabolic phenotyping in health and disease. Cell.

[25] Cambiaghi A, Pinto BB, Brunelli L (2017). Characterization of a metabolomic profile associated with responsiveness to therapy in the acute phase of septic shock. Sci Rep.

[26] Jaurila H, Koivukangas V, Koskela M (2020). (1)H NMR based metabolomics in human sepsis and healthy serum. Metabolites.

[27] Schmerler D, Neugebauer S, Ludewig K, Bremer-Streck S, Brunkhorst FM, Kiehntopf M (2012). Targeted metabolomics for discrimination of systemic inflammatory disorders in critically ill patients. J Lipid Res.

[28] To KKW, Lee K-C, Wong SSY (2016). Lipid metabolites as potential diagnostic and prognostic biomarkers for acute community acquired pneumonia. Diagn Microbiol Infect Dis.

[29] Ping F, Li Y, Cao Y (2021). Metabolomics analysis of the development of sepsis and potential biomarkers of sepsis-induced acute kidney injury. Oxid Med Cell Longev.

[30] Slupsky CM, Cheypesh A, Chao DV (2009). *Streptococcus pneumoniae* and *Staphylococcus aureus* pneumonia induce distinct metabolic responses. J Proteome Res.

[31] Mickiewicz B, Duggan GE, Winston BW, Doig C, Kubes P, Vogel HJ (2014). Metabolic profiling of serum samples by 1H nuclear magnetic resonance spectroscopy as a potential diagnostic approach for septic shock. Crit Care Med.

[32] Ferrario M, Cambiaghi A, Brunelli L (2016). Mortality prediction in patients with severe septic shock: a pilot study using a target metabolomics approach. Sci Rep.

[33] Rogers AJ, McGeachie M, Baron RM (2014). Metabolomic derangements are associated with mortality in critically ill adult patients. PLoS ONE.

[34] Nickler M, Ottiger M, Steuer C (2015). Systematic review regarding metabolic profiling for improved pathophysiological understanding of disease and outcome prediction in respiratory infections. Respir Res.

[35] Zeisel SH, Warrier M (2017). Trimethylamine N-Oxide, the microbiome, and heart and kidney disease. Annu Rev Nutr.

[36] Pierrakos C, Vincent J-L (2010). Sepsis biomarkers: a review. Crit Care.

[37] Hoerr V, Zbytnuik L, Leger C, Tam PPC, Kubes P, Vogel HJ (2012). Gram-negative and Gram-positive bacterial infections give rise to a different metabolic response in a mouse model. J Proteome Res.

[38] Smith CA, Want EJ, O’Maille G, Abagyan R, Siuzdak G (2006). XCMS: processing mass spectrometry data for metabolite profiling using nonlinear peak alignment, matching, and identification. Anal Chem.

[39] Scheltema RA, Jankevics A, Jansen RC, Swertz MA, Breitling R (2011). PeakML/mzMatch: a file format, Java Library, R Library, and tool-chain for mass spectrometry data analysis. Anal Chem.

[40] Gloaguen Y, Morton F, Daly R (2017). PiMP my metabolome: an integrated, web-based tool for LC-MS metabolomics data. Bioinformatics.

[41] Bengoechea JA, Bamford CGG (2020). SARS-CoV-2, bacterial co-infections, and AMR: the deadly trio in COVID-19?. EMBO Mol Med.

